# Mutant alleles of the *Caenorhabditis elegans rde-1* gene identified through chemical mutagenesis of an snRNA misprocessing reporter

**DOI:** 10.1093/g3journal/jkaf097

**Published:** 2025-05-05

**Authors:** Brandon M Waddell, Cheng-Wei Wu

**Affiliations:** Department of Veterinary Biomedical Sciences, Western College of Veterinary Medicine, University of Saskatchewan, Saskatoon, Canada, SK S7N 5B4; Department of Veterinary Biomedical Sciences, Western College of Veterinary Medicine, University of Saskatchewan, Saskatoon, Canada, SK S7N 5B4; Toxicology Centre, University of Saskatchewan, Saskatoon, Canada, SK S7N 5B3; Department of Biochemistry, Microbiology and Immunology, College of Medicine, University of Saskatchewan, Saskatoon, Canada, SK S7N 5E5

**Keywords:** snRNA, *C. elegans*, mutagenesis, argonaute, wormBase

## Abstract

The processing of small nuclear RNA post transcription involves endolytic cleavage of a 3′ tail to produce a mature transcript that is incorporated into the spliceosome to regulate RNA splicing. We previously reported in the *Caenorhabditis elegans* model several novel genetic regulators including those functioning in RNAi processing to be required for snRNA cleavage through a genome-wide RNAi screen using an in vivo snRNA misprocessing reporter. Here, we conducted a forward genetic screen using the mutagen ethyl methanesulfonate to screen for viable mutants that exhibit constitutive snRNA misprocessing. This screen generated three new recessive *rde-1* mutant alleles (*cww1, cww4, cww9*) identified via WGS SNP mapping, which encode the primary Argonaute protein involved in the processing of exogenous RNAi. The three *rde-1* alleles failed to complement each other and *rde-1(cww1)* which contains a premature stop codon in exon 3 also failed to be complemented by the classic *rde-1(ne219)* allele. We show that the three *rde-1* mutants display a varying degree of snRNA misprocessing reporter activation, but are all fully resistant to various RNAi that are known to cause larval arrest or an abnormal vulva phenotype. Thus, the screen has reinforced a connection between RNAi processing and snRNA cleavage and generated mutants that are useful for future studies of the *rde-1* Argonaute gene.

## Introduction

The small nuclear RNA (snRNA) transcripts are noncoding RNA that interact with small nuclear ribonucleoproteins to form an active spliceosome that is required for pre-mRNA splicing in eukaryotes (Wilkinson *et al.* 2020). After transcription by RNA polymerase II, snRNA transcripts possess an extended 3′ end that requires endolytic cleavage before its incorporation into the spliceosome. The Integrator was discovered in 2005 as the protein complex required for 3′ snRNA cleavage, a process that has thus far been found to be conserved across metazoans (Baillat *et al.* 2005; Ezzeddine *et al.* 2011; Kapp *et al.* 2013; Gómez-Orte *et al.* 2019; Wu *et al.* 2019). Disruption of Integrator function results in the accumulation of misprocessed snRNA transcript that perturbs RNA splicing, but can also cause alteration to gene expression via its other function in regulating RNA polymerase pause-release (Gardini *et al.* 2014; Skaar *et al.* 2015; Oegema *et al.* 2017).

We recently developed in the *Caenorhabditis elegans* model an in vivo GFP-based snRNA misprocessing reporter as a biomarker for Integrator malfunction and performed a genome-wide RNAi screen for novel genetic regulators (Waddell and Wu 2024). In that study, we identified 47 genes when knocked down by RNAi caused an increase in GFP activation as an indicator of snRNA misprocessing, these include various subunits of the Integrator and genes required for translation and nuclear organization (Waddell and Wu 2024). The vast majority of the genes identified in the RNAi screen were essential, indicating that snRNA processing is indispensable for life. However, a small number of nonessential genes were also uncovered in the RNAi screen, all of which function within the RNAi pathway including *mut-16* (MUTator), *alg-4* (Argonaute Like Gene), and *rde-4* (RNAi DEfective) (Tabara *et al.* 1999; Conine *et al.* 2010; Zhang *et al.* 2011). We also uncovered essential genes related RNAi function including *csr-1* (Chromosome-Segregation and RNAi deficient) and *dcr-1* (DiCer Related), that, when knocked down by RNAi activated the snRNA misprocessing reporter.

To explore the possibility that mutation to nonessential genes can trigger snRNA processing defects, we performed a forward genetic screen with the chemical mutagen ethyl methanesulfonate (EMS) in *C. elegans*. The rationale for this approach is that the recovery of a viable mutant from an EMS screen would likely indicate a mutation to a nonessential gene, or possibly a hypomorphic mutation to an essential gene. Both prospects would be of interest as they would indicate a condition where cells are viable despite the persistence of snRNA misprocessing. Here, we report the identification of three new mutant alleles of *rde-1* gene that encode the primary *C. elegans* Argonaute protein required for assembly of the RISC complex (Tabara *et al.* 1999; Parrish and Fire 2001). We find that all three *rde-1* alleles activate the snRNA misprocessing reporter to varying degrees but are all highly resistant to RNAi, indicating that the nature of these mutations interferes with *rde-1*'s function in RNAi processing.

## Materials and methods

### *Caenorhabditis elegans* strains and maintenance

*Caenorhabditis elegans* were cultured with standard conditions at 20°C on nematode growth media (NGM) plates seeded with *E. coli*  OP50 as described (Brenner 1974). The strains used this in study were as follow: N2 bristol wildtype, MWU3  *cwwIs1[C47F8.9p::C47F8.9::GFP; myo-2p::tdTomato]*, CB4856 Hawaii isolate, MWU44  *rde-1(cww1); cwwIs1*, MWU45  *rde-1(cww1),*  MWU56  *rde-1(cww4); cwwIs1,*  MWU57  *rde-1(cww4),*  MWU66  *rde-1(cww9)*; *cwwIs1*, MWU67  *rde-1(cww9)*, WM27  *rde-1(ne219).*

### EMS mutagenesis and WGS mapping

The protocol for EMS mutagenesis was as previously described by (Brenner 1974) with minor modifications. Briefly, a population of ∼2,000 MWU3 were synchronized and grown to the L4 stage followed by mutagenesis by incubating with 50 mM of EMS for 4 h on an orbit rotator at room temperature (∼20°C). Following mutagenesis, worms were then washed 4 times with M9 by gravity settling before being transferred to a 10-cm NGM agar plate seeded with NA22  *E. coli* to recover for 2 h. Post recovery, worms were collected in M9 buffer and ∼50–75 worms were plated on each of thirty 10-cm NA22 seeded plates and incubated at 16°C overnight. The next morning, P_0_ adults were removed from all plates by gently washing with M9 buffer and discarded leaving behind the F_1_ eggs which were allowed to grow for 3 days at 20°C to reach adulthood and allowed to lay F_2_ eggs overnight. Once F_2_ eggs were laid, the F_1_ adults were first manually screened for GFP activation with an Olympus SZX61 fluorescent stereomicroscope. Afterwards, the F_1_ adults were removed by gentle washing with M9 buffer. The F_2_ generation was allowed to grow for 2 days to reach adulthood, and the thirty plates were manually screened again for GFP activation.

The isolated mutants were backcrossed 4 times with MWU3 to remove background mutations followed by crossing with males from the CB4856 Hawaiian polymorphic strain for SNP mapping using methods described by (Doitsidou *et al.* 2010). After mating, 50 GFP positive F_2_ hermaphrodites were singled out and allowed to self-reproduce for 1–2 generations before pooling the worms for DNA extraction. Genomic DNA was extracted with a PureLink Genomic DNA Mini Kit (K192001) followed by library construction with the Nextera Flex Library Prep Kit (Illumina, Cat# 20018704 and 20027213) and sequencing on a NextSeq 550 using a Mid Output kit (Illumina, Cat#20024904). Reads were extracted and trimmed using the Illumina Generate FASTQ BaseSpace pipeline (version 1.37.0) and the mutation loci were mapped by the MiModD (Mutation identification in Model organism genomes using Desktop PCs) protocol using the WS220/ce10 reference genome. The MiModD software used is available in https://sourceforge.net/projects/mimodd/ and the workflow used are described in full at https://mimodd.readthedocs.io/en/latest/.

### RNAi experiments

RNAi experiments were previously described in detail (Chomyshen *et al.* 2022). Briefly, synchronized L1 wildtype, *rde-1(cww1)*, *rde-1(cww4)*, or *rde-1(cww9)* worms were grown on NGM agar plates containing 50 µg mL ^−1^ carbenicillin and 100 μg mL ^−1^ of isopropyl β-D-thiogalactopyranoside (IPTG) seeded with *HT115(DE3) E. coli* expressing empty vector (EV, L4440 plasmid), *cdl-1* (Cell Death Lethal), *ifg-1* (Initiation Factor 4G), *inf-1* (INitiation Factor), or *npp-6* (Nuclear Pore complex Protein) RNAi clones. Worms were grown for ∼55 h at 20°C to reach the first day of adulthood followed by imaging directly on the agar plate to assess body length and vulva protrusion phenotypes. All RNAi clones were verified through Sanger sequencing.

### Microscopy

To image *C. elegans* in a microscope slide, worms at the L4 stage were anesthetized by mounting on a 2% agar plate containing 5 µL of 2% sodium azide dissolved in M9 followed by fluorescent imaging with a Zeiss Axioskop 50 fitted with a Retiga R3 camera. To image *C. elegans* in the agar plate, synchronized worms were grown for 55 h to reach adulthood at 20°C and directly imaged on the agar plate using an Olympus SZX61 fluorescent stereomicroscope mounted with a Retiga R3 camera. Body size measurements were performed in ImageJ using the measure function and converted to millimeter units using a micrometer microscope calibration slide. For imaging *C. elegans* in a microplate well, ∼ 100 F_1_ worms from the *rde-1(cww1)* × *rde-1(ne219)* cross were picked into a microplate well containing 100 µL of M9 with 0.5% sodium azide and imaged using the Cytation-5 multimode system.

### Complementation assays

To complement the mutations isolated, 15 males from one strain are placed in the center of a 6-cm agar plate containing a 5 µL drop of *E. coli*  OP50 with 5 hermaphrodites from another strain and allowed to mate. After 3 days, F_1_ progenies from the cross were assessed for GFP activation and imaged on a microscope slide or 96-well microplate as indicated in the figure legend.

### RNA extraction and qPCR

Protocols for RNA extraction and qPCR are previously described (Waddell and Wu 2024). Briefly, RNA from synchronized 1 day old wildtype, *rde-1(cww1)*, or *rde-1(ne219)* worms was extracted using the Purelink RNA mini kit (ThermoFisher, 12183020). The extracted RNA was treated with DNAseI (ThermoFisher EN0521) followed by cDNA library construction with the Invitrogen Mutiscribe reverse transcriptase system (ThermoFisher, 4311235). Gene expression was measured with qPCR using a QuantStudio 3 system with primers previously reported (Waddell and Wu 2024). Gene expression was normalized to the housekeeping gene *cdc-42.*

### Statistical analyses

The GraphPad Prism software (V8.4.3) was used to create graphical data and perform statistical analysis. Student's *t*-test was used to compare between 2 groups, One-way ANOVA with the Dunnett test was used for multiple comparisons, and the Chi-square test was used to test for contingency data.

## Results and discussion

### Genetic screen design for snRNA misprocessing mutants

**Fig. 1.**
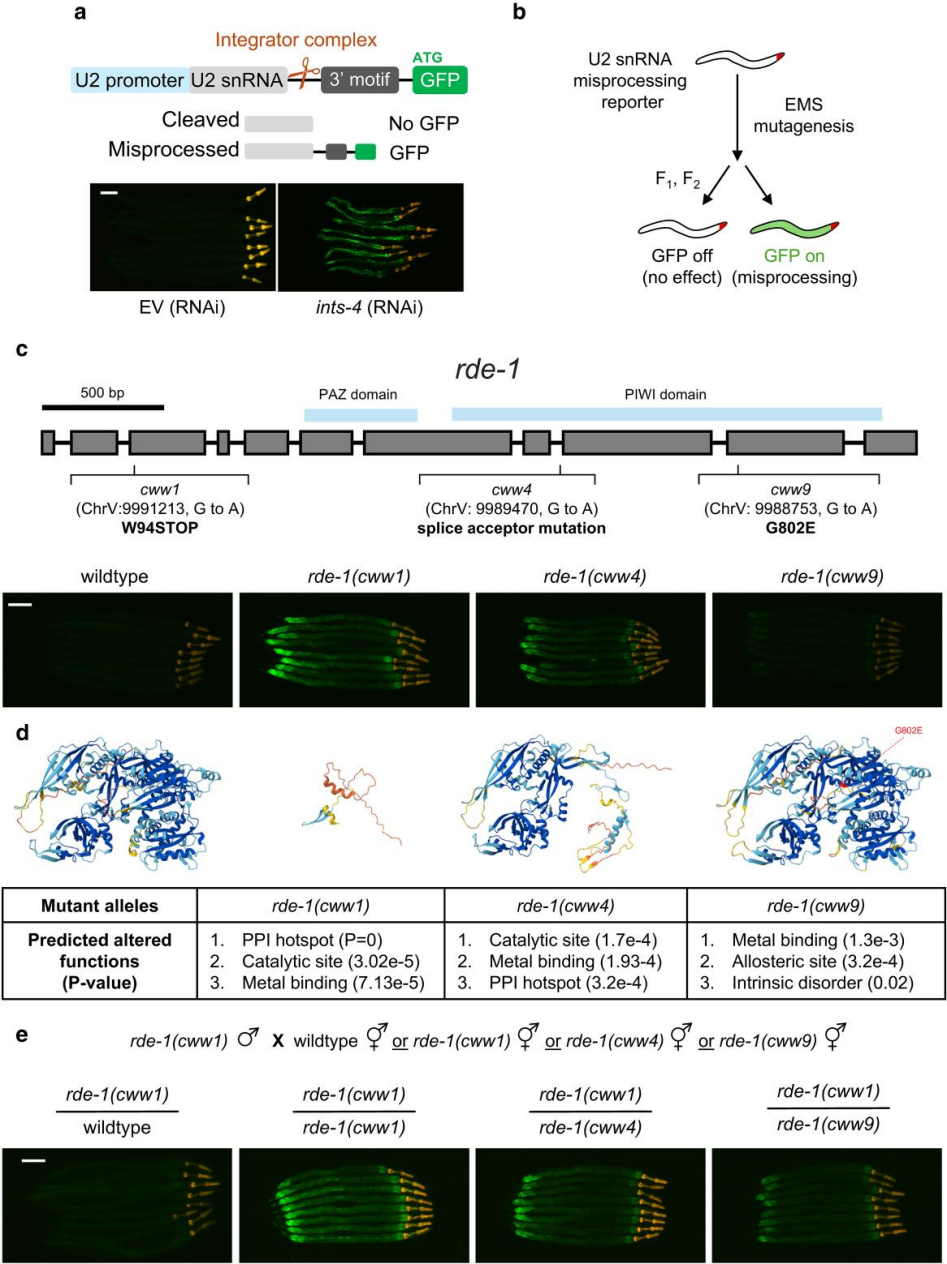
EMS mutagenesis identification of new *rde-1* mutant allele. a) Schematic of the GFP based snRNA misprocessing reporter. Fluorescent micrographs show the in vivo snRNA misprocessing strain fed with EV or *ints-4* RNAi. b) Outline of EMS mutagenesis workflow to identify mutants that activate the snRNA misprocessing reporter. c) The *rde-1* gene locus is marked with the PAZ and PIWI domain along with the location of the 3 mutations identified from the EMS screen. Representative fluorescent micrographs of worms expressing the snRNA misprocessing reporter in wildtype and the 3 isolated *rde-1* mutants are shown. The *rde-1* sequence and domain information was retrieved from Wormbase (Sternberg *et al.* 2024). d) AlphaFold3 structural and MutPred2 functional prediction of RDE-1 protein variants produced by the mutant alleles. e) Complementation assay showing the F_1_ offspring produced by crossing of male *rde-1(cww1)* mutants into wildtype, *rde-1(cww1)*, *rde-1(cww4)*, or *rde-1(cww9)* hermaphrodite. Microscopic images were taken by mounting worms on a glass slide and the scale bar indicates 100 µm.

To complement our recent study where we performed a genome-wide RNAi screen to identify genes required for snRNA processing in *C. elegans*, we carried out an EMS screen to search for viable mutants that exhibit snRNA processing defects. As previously described, we created a strain of *C. elegans* that expresses a stably integrated construct of the U2 snRNA transcript (C47F8.9) tagged with GFP downstream of the 3′ motif that serves as the signal for Integrator cleavage (Waddell and Wu 2024). Under normal conditions, GFP fluorescence is absent as Integrator cleavage near the 3′ motif terminates the GFP transcript. Upon Integrator disruption such as RNAi knockdown of Integrator subunit-4 (*ints-4*), cleavage failure results in transcriptional read-through of the reporter that extends the open reading frame to include the GFP transcript to produce fluorescence (Fig. 1a). Using this reporter strain, we mutagenized ∼ 2,000 L4 P_0_ worms and visually screened for F_1_ and F_2_ progenies that activate the GFP reporter (Fig. 1b).

### New alleles of *rde-1* mutation

We did not isolate any mutants when screening the F_1_ population for potential dominant mutations; however, we isolated 3 independent F_2_ recessive mutants that display varying degrees of GFP activation above the basal expression observed in the wildtype. We first backcrossed these 3 strains 4 times to the wildtype reporter strain followed by crossing with the polymorphic Hawaiian *C. elegans* isolate (CB4856) and carried out whole genome sequencing followed by SNP mapping as described previously (Doitsidou *et al.* 2010). SNP analysis mapped mutation to chromosome V for all 3 mutants, with each strain carrying a unique mutation to the *rde-1* gene. The *rde-1(cww1)* allele carries a premature stop codon mutation at amino acid 94 (W94X) and exhibits the brightest GFP fluorescence (Fig. 1c). Alphafold3 prediction shows that this mutation produces a severely truncated RDE-1 protein with an absent PAZ and PIWI domain required for RNA binding and catalytic function (Fig. 1d) (Abramson *et al.* 2024). Prediction analysis with MutPred2 indicates that this mutation would alter the protein-protein interaction (PPI), catalytic site, and metal binding properties compared with the wildtype RDE-1 protein (Fig. 1d) (Pejaver *et al.* 2020).

Mutations within the PIWI domain were found for *rde-1(cww4)* and *rde-1(cww9)*. The *rde-1(cww4)* strain shows moderate GFP activation and carries a G to A mutation to the 3′ splice site acceptor sequence of intron 8. This mutation alters the conserved AG dinucleotide at the end of the intronic sequence which is known to block splice-site usage by the P-complex spliceosome, that can alter the transcript reading frame (Horowitz 2012; Wilkinson *et al.* 2020). A predicted outcome of this mutation is the retention of intron 8 due to the loss of 3′ splice site acceptor, which would result in a premature stop codon due to the inclusion of intron 8 within the *rde-1* reading frame (Fig. 1d). The predicted structure of this protein retains the PAZ domain, but similar to the *cww1* allele, this mutation is predicted to alter the PPI, catalytic site, and metal binding of RDE-1. Meanwhile, *rde-1(cww9)* introduces a missense mutation from glycine to glutamic acid at amino acid position 802 and exhibits a weak but consistent activation of GFP in the posterior region of the intestine (Fig. 1c). Alphafold3 prediction of this mutant protein only shows slight structural variations compared with the wildtype RDE-1 (Supplementary Fig. 1). However, analysis of the G802E mutation with MutPred2 predicts various protein alterations, including increases in intrinsic disorder, loss of catalytic site at R800, and altered DNA binding (Fig. 1d) (Pejaver *et al.* 2020).

To further confirm the 3 mutants carry mutations to the *rde-1* gene, we next performed complementation testing by crossing male *rde-1(cww1)* into hermaphrodite wildtype and the 3 *rde-1* mutants (Fig. 1e). Conventionally, complementation testing would be performed prior to WGS for allele identification; however, modern improvements to sequence capacity have made it possible to analyze several samples at once while still achieving sufficient mapping coverage for accurate variant detection. Worms heterozygous for the *rde-1(cww1)* allele lose their GFP activation, as expected given the mutation is recessive. Both the *rde-1(cww4)* and *rde-1(cww9)* alleles fail to complement *rde-1(cww1)*, strengthening the evidence that these 3 mutants carry a mutation to the same gene. Interestingly, the biallelic *rde-1(cww1)/rde-1(cww9)* mutation exhibits visibly brighter GFP fluorescence compared with the *rde-1(cww9)* homozygote mutant, suggesting that the W94X mutation in the *cww1* allele strengthens snRNA misprocessing of the G802E mutation.

### *rde-1* requirement in snRNA processing

**Fig. 2.**
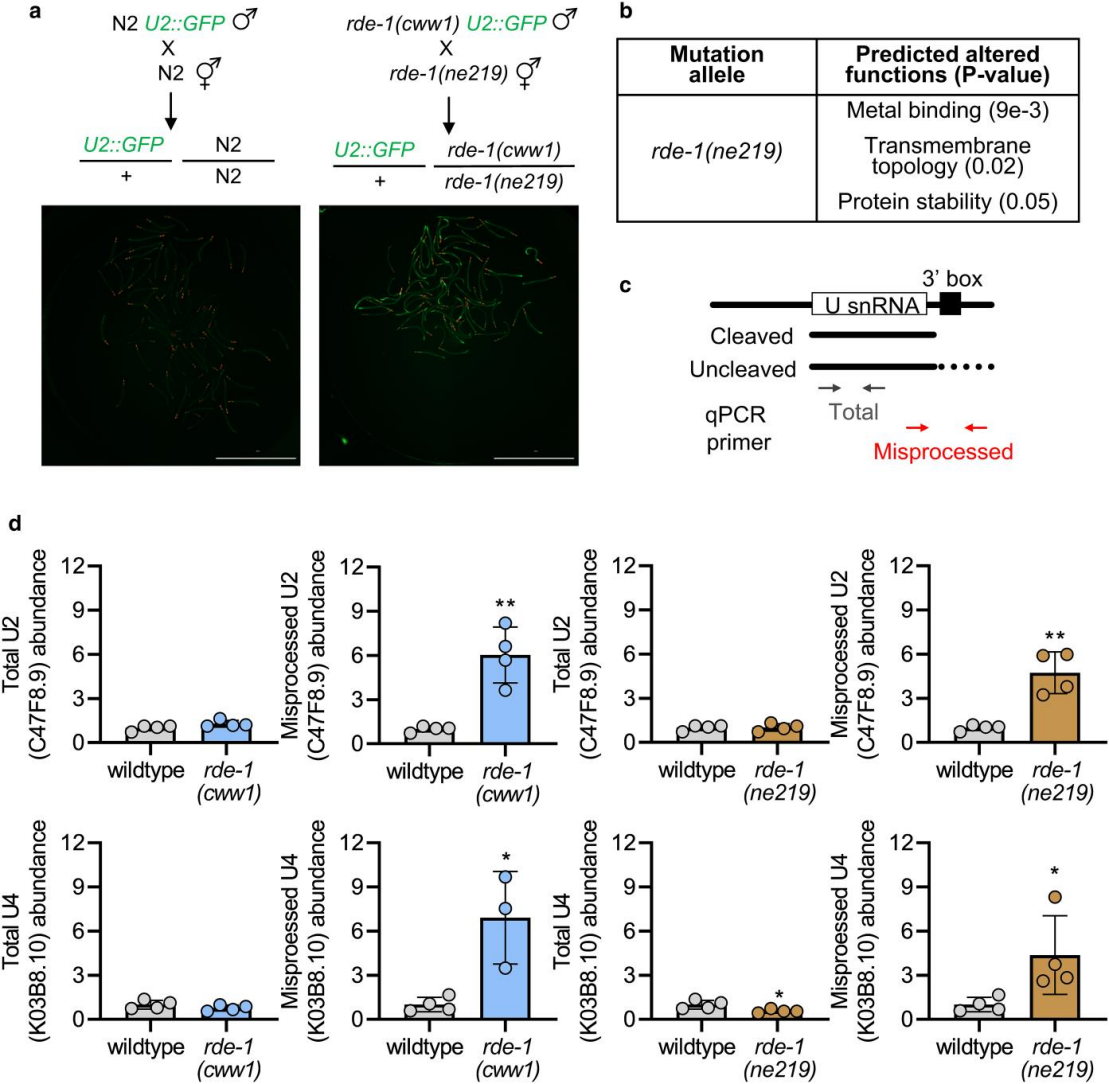
Endogenous misprocessing of snRNA in *rde-1* mutants. a) Complementation assay showing the F_1_ offspring produced by crossing male *rde-1(cww1)* mutants into *rde-1(ne219)* hermaphrodites. An N2 cross illustrating the basal GFP levels of the snRNA misprocessing reporter in heterozygotes is shown in parallel. Microscopic images were taken with worms aliquoted in a 96-well microplate, and the scale bar indicates 2000 µm. b) MutPred2 prediction of altered functions of the *rde-1(ne219)* mutant. c) Schematic of qPCR primers used to measure total or misprocessed snRNA transcripts. d) Relative levels of total or misprocessed U2 (C47F8.9) and U4 (K03B8.10) transcripts in wildtype, *rde-1(cww1),* or *rde-1(ne219)* background. *N* = 3–4 samples with each sample containing ∼500 worms.*P < 0.05, **P < 0.01 as determined by the student's *t*-test.

Several variants of the *rde-1* mutants have been isolated to date, with the most frequently used allele being the *rde-1(ne219)* mutant isolated in the 1999 landmark study that carries an E414 K mutation within the PAZ domain of the gene (Tabara *et al.* 1999). We next performed a complementation test by crossing male *rde-1(cww1)* with the hermaphrodite *rde-1(ne219)* mutant. We chose to focus on *rde-1(cww1)* as it exhibited the strongest activation of the snRNA misprocessing reporter. Since the *rde-1(ne219)* mutant does not express the snRNA misprocessing reporter, we also crossed the wildtype reporter strain with N2 to obtain the basal GFP fluorescence of the reporter when expressed as a heterozygote. We found that *rde-1(ne219)* also failed to complement *rde-1(cww1)*, as all the F_1_ progenies biallelic for *rde-1(cww1)/rde-1(ne219)* show strong GFP fluorescence (Fig. 2a). This result further supports *rde-1* mutation as the cause for activation of the snRNA misprocessing reporter. Alphafold3 prediction shows that *rde-1(ne219)* exhibits minor structural variation compared with the wildtype (Supplementary Fig. 1). MutPred2 analysis shows that the E414 K mutation of the *rde-1(ne219)* allele predicts alterations to the metal binding properties of RDE-1 but also exhibits changes to transmembrane topology and protein stability (Fig. 2b).

Next, to confirm mutations to *rde-1* alter snRNA processing, we first crossed out the misprocessing reporter in the *rde-1(cww1)* and performed qPCR using a pair of primers that detect total and misprocessed levels of U2 and U4 (K03B8.10) snRNA (Fig. 2c). The removal of the reporter eliminates the possibility that the primer detects the transgenic expression of the U2 snRNA and will only measure the endogenous transcripts. We found that in both the *rde-1(cww1)* and *rde-1(ne219)* mutants, there were no changes to total U2 or U4 snRNA levels; however, both mutants show elevated levels of misprocessed U2 and U4 transcripts (Fig. 2d). This indicates that mutations to *rde-1* affect the processing of multiple U snRNA, and the effects are not limited to the processing of the U2 snRNA. The degree of misprocessing triggered by mutation to *rde-1* is comparable to what we previously reported in *csr-1* mutants (Waddell and Wu 2024). However, they are relatively minor compared with when Integrator subunits are depleted directly, which can cause a > 100-fold increase in misprocessed snRNA (Waddell *et al.* 2025). This variability in the degree of snRNA misprocessing may partially explain why *rde-1* mutants are viable despite exhibiting constitutive snRNA processing defects. This could suggest that there is a threshold for which accumulation of misprocessed snRNA triggers embryonic lethality, which is not reached in *rde-1* mutants that show relatively minor degrees of processing error compared with direct Integrator malfunction.

### *rde-1* mutations confer loss of function in RNAi processing

**Fig. 3.**
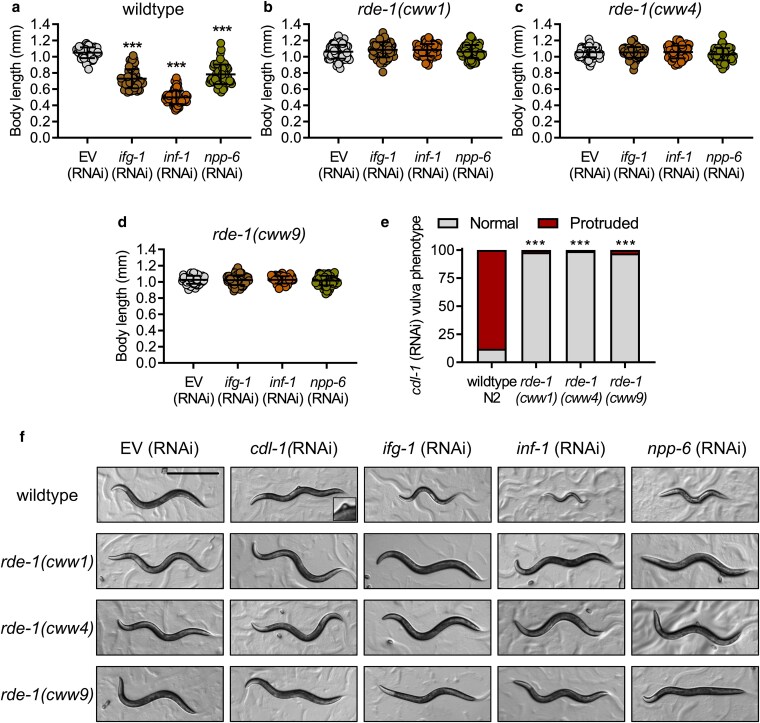
New *rde-1* mutant alleles are RNAi resistant. Body length measurement of *C. elegans* after feeding of EV, *ifg-1*, *inf-1*, and *npp-6* dsRNA in a) wildtype, b) *rde-1(cww1)*, c) *rde-1(cww4)*, and d) *rde-1(cww9)* worms. *N* = 67–83 worms scored in each condition. e) Percentage of *C. elegans* exhibiting the protruded vulva phenotype after *cdl-1* (RNAi) in wildtype, *rde-1(cww1)*, *rde-1(cww4)*, or *rde-1(cww9)* worms. *N* = 240–325 worms scored per condition. f) Representative micrographs illustrating wildtype and *rde-1* mutant *C. elegans* strain 55 h after RNAi feeding from L1. The protruded vulva phenotype is magnified in the wildtype + *cdl-1(RNAi)* image. Microscopic pictures were taken with worms directly on agar plates, and the scale bar indicates 500 µm. ***P < 0.001 as determined by One-way ANOVA in a) and by the Chi-square test in e).

The hallmark phenotype of *rde-1* loss of function mutants is resistance to RNAi, caused by a defect in the initial processing of exogenous dsRNA (Tabara *et al.* 1999; Parrish and Fire 2001). Given that the 3 *rde-1* alleles isolated in this study show varying degrees of snRNA misprocessing reporter activation, we next use RNAi penetrance as a way to assess whether these mutations are strong or partial loss of function variants. We fed the worms dsRNA to knockdown genes that were previously shown to cause high penetrance of larval arrest (*ifg-1*, *inf-1*, *npp-6*), or vulva protrusion (*cdl-1*) to evaluate the function of the 3 *rde-1* mutants (Kodama *et al.* 2002; Pettitt *et al.* 2002; Chomyshen *et al.* 2022; Waddell and Wu 2024). Knockdown of *ifg-1, inf-1*, and *npp-6* all led to a significant decrease in body size of the wildtype worm; however, all 3 *rde-1* mutants were completely unaffected by these RNAi effects (Fig. 3, a–d and f). Knockdown of *cdl-1,* which functions to process histone biosynthesis, caused vulva protrusion in 88% of wildtype worms but had next to no effect (1–3%) on the 3 *rde-1* mutants (Fig. 3e, f). These results indicate that all 3 *rde-1* mutants have a strong loss of function to RNAi processing. While this was expected for *rde-1(cww1)* given that it is prematurely terminated in exon 3, the same degree of RNAi resistance observed for *rde-1(cww4)* and *rde-1(cww9)* highlights the essential role of the PIWI domain in the function of *rde-1* for RNAi processing. These loss of function phenotype is also consistent with MutPred2 prediction of altered RDE-1 functions. It remains to be determined why the 3 *rde-1* mutants show varying degrees of snRNA misprocessing reporter activation, as this could indicate that *rde-1* influences snRNA cleavage in a mechanism that is distinct from its role in forming the RISC complex for RNAi silencing (Tabara *et al.* 1999, 2002).

## Conclusion

In summary, through the use of a forward genetic screen to search for genes required for snRNA processing, we uncovered 3 new loss of function or hypomorphic alleles of the *rde-1* gene encoding the primary Argonaute protein required for triggering the RNAi response in *C. elegans*. This study complements our recent reverse genetic screen that identified knockdown of *dcr-1* and *rde-4* activates the snRNA misprocessing reporter, with both genes functioning with *rde-1* in the initial processing of exogenous dsRNA (Tabara *et al.* 2002). However, in that study, we failed to identify *rde-1* in the RNAi screen, potentially due to the ineffectiveness of using RNAi to deplete *rde-1* which would impair exogenous dsRNA processing to create a feedback loop that would diminish RNAi penetrance. As such, this forward genetic screen uncovered a role for *rde-1* in snRNA processing that would otherwise be missed in the RNAi screen.

We recognize a limitation of this study is that we have not identified the functional mechanism driving *rde-1*'s role in snRNA processing. A future direction will be to investigate whether *rde-1* influences snRNA processing in a similar mechanism as *csr-1,* which we recently reported to be required for maintaining the proper expression of Integrator subunit proteins (Waddell and Wu 2024). Overall, while CRISPR/Cas9 has made it relatively straightforward to create null mutants of nonessential genes, we believe that the *rde-1* mutants discovered through our forward genetic screen, which produced an assortment of variants including premature stop mutation, splice site acceptor mutation, and missense mutation, are informative to understanding the functions of different domains of the *rde-1* gene. As such, these alleles will be broadly useful to the *C. elegans* community that continues to study the role of this Argonaute protein in emerging contexts such as innate immune response to viral infection (Félix *et al.* 2011).

## Supplementary Material

jkaf097_Supplementary_Data

## Data Availability

All datasets supporting this manuscript are presented within the article. Strains containing the new alleles will be deposited to the CGC and are also available upon request. Supplemental material available at G3 online.
