# Supplementary material for: Mutant alleles of the Caenorhabditis elegans rde-1 gene identified through chemical mutagenesis of an snRNA misprocessing reporter
Source: G3 (Bethesda). 2025 May 5;15(7):jkaf097. doi: 10.1093/g3journal/jkaf097 (PMC12239602; doi:10.1093/g3journal/jkaf097)
Supplement: jkaf097_Supplementary_Data [file jkaf097_supplementary_data.zip › Supplemental_Figure_Legend_G3-2025-405830.docx]

**Supplemental Figure Legend**

**Figure S1**. **Predicted structures of *rde-1* mutant variants.** Predicted RDE-1 structures encoded by the *rde-1(cww9)* (orange) and *rde-1(ne219)* (green) mutants superimposed on the wildtype protein (black).
